# Genetic mutations in nonsyndromic deafness patients of Chinese minority and han ethnicities in Yunnan, China

**DOI:** 10.1186/1479-5876-11-312

**Published:** 2013-12-17

**Authors:** Feng Xin, Yongyi Yuan, Xiaoming Deng, Mingyu Han, Guojian Wang, Jiandong Zhao, Xue Gao, Jun Liu, Fei Yu, Dongyi Han, Pu Dai

**Affiliations:** 1 Department of Otolaryngology and Genetic Testing Center for Deafness, Chinese PLA General Hospital, Beijing 100853, P.R. China; 2 Department of Otorhinolaryngology, Hainan Branch of PLA General Hospital, Sanya, P.R. China; 3 Department of Otorhinolaryngology, First Hospital, Yunnan 650032, P.R. China; 4 Department of Otolaryngology, The Second Artillery General Hospital, 16# Xin Wai Da Jie, Beijing 100088, P.R. China

**Keywords:** Genetic mutations, Nonsyndromic deafness, Minority, Han, Yunnan

## Abstract

**Background:**

Each year in China, 30,000 babies are born with congenital hearing impairment. However, the molecular etiology of hearing impairment in the Yunnan Province population where more than 52 minorities live has not been thoroughly investigated. To provide appropriate genetic testing and counseling to these families, we investigated the molecular etiology of nonsyndromic deafness in this population.

**Methods:**

Unrelated students with hearing loss (n = 235) who attended Kunming Huaxia secondary specialized school in Yunnan enrolled in this study. Three prominent deafness-related genes, *GJB2*, *SLC26A4* and *mtDNA 12S rRNA*, were analyzed. High-resolution temporal bone computed tomography (CT) scan examinations were performed in 100 cases, including 16 cases with *SLC26A4* gene variants, and 37 minorities and 47 Han cases without any *SLC26A4* gene mutation.

**Results:**

The *GJB2* mutation was detected in 16.67% (7/42) of minority patients and 17.62% (34/193) of Chinese Han patients (P > 0.05). 235delC was the hotspot mutation in nonsyndromic hearing loss (NSHL) patients, whereas 35delG was not found. The 431_450del19 mutation was detected for the first time in Han NSHL patients, which resulted in a premature stop codon and changed the protein. The *SLC26A4* mutation was found in 9.52% (4/42) of minority patients and 9.84% (19/193) of Han Chinese patients (P > 0.05). The frequencies of *mtDNA 12S rRNA* mutation in minority and Han Chinese patients were 11.90% (5/42) and 7.77% (15/193; P > 0.05), respectively. Sixteen (16/23, 69.57%) patients with *SLC26A4* mutations received temporal bone CT scan, and 14 patients were diagnosed with enlarged vestibular aqueducts (EVAs); the other 2 patients had normal inner ear development. The ratio of EVA in the minorities was 14.63% (6/41).

**Conclusions:**

In this study, a total of 35.74% deaf patients showed evidence of genetic involvement, based on either genetic screening or family history; 17.45%, 9.79%, and 8.51% of the patients were determined to have inherited hearing impairment caused by *GJB2*, *SLC26A4*, and mtDNA 1555A > G mutations. There was no significant difference in deafness associated gene mutational spectrum and frequency between the Yunnan minority and Han patients.

## Introduction

Hearing impairment is the most common neurosensory disorder in humans, with an incidence of 1 in 300 to 1 in 1,000 children [1-3] and approximately half of all cases are caused by genetic defects. *GJB2*, *SLC26A4* and mitochondrial DNA (*mtDNA*) *12S rRNA* are thought to be the most common causes of nonsyndromic autosomal recessive deafness in Asian people. China is a large country with 1.3 billion people of 56 ethnicities. For effective genetic testing and accurate counseling, comprehensive genetic analysis in deaf patients in most Chinese regions had been performed. However, little is known of the Yunnan Province, located southeast of mainland China, where 52 nationalities—including Han and 51 minorities—live together. Yunnan Province, which comprises more than 52 ethnic groups, is an important cradle of civilization. Among the 52 Chinese ethnicities, the 16 native to Yunnan are: Yi, Bai, Hani, Dai, Lisu, Lahu, Wa, Naxi, Jingpo, Brown, Prattm, Achang, Nu, Jino, Deang and Dulong. Data released by the Yunnan province Disabled Persons’ Federation showed that 7.47% (477/6,383) of handicapped out-of-school children suffered from hearing impairment.

Worldwide, the most common cause of nonsyndromic autosomal recessive hearing loss is a mutation in connexin 26, a gap-junction protein encoded by the *GJB2* gene [4-11]. Defects in *SLC26A4*, which encodes the anion (chloride/iodide, chloride/bicarbonate) transporter Pendrin, can cause nonsyndromic DFNB4 deafness with enlargement of the vestibular aqueduct (EVA) and Pendred syndrome [12,13]. A *SLC26A4* mutation is the second most common cause of deafness in China [4]. Although the majority of cases of hereditary hearing loss are caused by nuclear gene defects, it has become clear that mutations in mtDNA can also cause nonsyndromic hearing loss. The best-studied mutations related to aminoglycoside susceptibility are A1555G and C1494T in the *mtDNA 12S rRNA* gene [14-16]. Nonsyndromic inherited hearing impairment caused by mutations in *GJB2*, *SLC26A4*, or *mtDNA 12S rRNA* typically account for 33.8% of the cases of deafness in areas of China [17].

Connexin 26, a gap junction protein encoded by the *GJB2* gene, is the most common molecular defect in nonsyndromic autosomal recessive deafness in China [4-11]. The *GJB2* gene mutational site exhibits racial differences: the high-incidence mutational site is 30_35delG in Caucasians, 167delT in Jews, and 233_235delC in Japanese. The molecular epidemiological investigation of deafness in China shows that *GJB2* 235delC has a high prevalence in Chinese deaf populations. Among recessive mutations, 35delG is the most frequent in Caucasians, 167delT in Ashkenazi Jews, 235delC in East Asians, and R143W in Africans, suggesting the existence of founder effects in different ethnic groups [7,14-16,18-26].

In the present study, we comprehensively analyzed three prominent deafness-related genes (i.e., *GJB2*, *SLC26A4*, *mtDNA 12S rRNA*) in 235 patients, including 45 minority patients from unrelated families in the Yunnan province who experienced early onset, nonsyndromic hearing impairment, to investigate the molecular etiology of hereditary hearing loss in this region. Detailed genotype and phenotype analyses were performed to provide effective risk assessment and genetic counseling for hearing loss patients and their families in this area.

## Materials and Methods

### Patients and DNA samples

Unrelated hearing loss patients (n = 235) from Huaxia secondary special education school in Yunnan province were enrolled in this study. This cohort of patients consisted of 133 males and 102 females from 17 to 23 years old, with an average age of 17.26 ± 2.12 years. Huaxia school is the only secondary special education school for the disabled in Yunnan, whose students come from all over the province. The ethnic minority composition in Huaxia school (30.2%) is consistent with that of the province’s. Ethnically, the patients included 193 Han (82.1%), 13 Bai (5.5%), 5 Dai (2.1%), 7 Yi (3.0%), 2 Hani (0.9%), 3 Hui (1.3%), 1 Jingpo (0.4%), 2 Lahu (0.9%), 2 Lisu (0.9%), 5 Nazi (2.1%), 1 Wa (0.4%), and 1 Yao (0.4%) Chinese.

The study protocol was performed with the approval of the ethnicity committee of the Chinese PLA General Hospital. Informed consent was obtained from all subjects prior to blood sampling. Parents were interviewed with regard to age of onset, family history, health of the mother during pregnancy and the clinical history of the patient, including infection, possible head or brain injury, and the use of aminoglycoside antibiotics. All subjects had moderate to profound bilateral sensorineural hearing impairment on audiograms. Careful medical examinations resulted in two patients being diagnosed with Waardenburg syndrome. DNA was extracted from the peripheral blood leukocytes of 235 patients with nonsyndromic hearing loss and 200 region- and race-matched controls with normal hearing using a commercially available DNA extraction kit (Watson Biotechnologies Inc, Shanghai, China).

### Mutational analysis

The coding exons plus approximately 50–100 bp of the flanking intron regions of *GJB2*, *mtDNA 12S rRNA* and *SLC26A4* were amplified by polymerase chain reaction (PCR), then sequenced using the Big Dye sequencing protocol in all patients. The sequence results were analyzed using an ABI 3100 DNA sequencing machine (Applied Biosystems, Foster City, CA) and ABI 3100 Analysis Software v.3.7 NT, according to the manufacturer’s protocol. Patients with monoallelic *GJB2* coding region mutations were further tested for *GJB2* IVS1 + 1G > A mutation or defects in *GJB2* exon 1 and its basal promoter.

For all 235 patients, 20 *SLC26A4* exons were individually sequenced starting from the frequently mutated exons 7, 8, 10 and 19. Patients with a single mutation on the above four exons were screened for the other exons in *SLC26A4*.

Two hundred controls with normal hearing were sequenced to determine the presence of mutations in the *GJB2* and *mtDNA 12S rRNA* polymorphisms. In addition, all controls were screened for *SLC26A4* mutations by denaturing high-pressure liquid chromatography (DHPLC) followed by sequencing.

### CT scan and thyroid examination

A total of 100 patients, including 16 cases with variants in *SLC26A4*, 37 minority patients and 47 Han cases without any mutations or variants in *SLC26A4*, were examined using temporal bone CT scans to diagnose EVA. EVA diagnosis was based on findings of a diameter of >1.5 mm at the midpoint between the common crus and the external aperture [27] or other inner ear malformations to evaluate Pendred syndrome. Thyroid ultrasound scans and thyroid hormone level determination was performed in patients with *SLC26A4* mutations or variants. These procedures were performed at the Frist Hospital of Yunnan province, China. As perchlorate discharge testing is not a general clinical practice in China, it was not used in this study.

### Statistical method

The statistical analysis was performed using SPSS 15.0 software (statistica package for the Social Sciences). The intergroup difference in frequency was compared using the two-tailed chi-square test. A P-value less than 0.05 was considered statistically significant.

## Results

### GJB2

Our sequence analysis of the *GJB2* gene indicated that 34 patients carried two confirmed pathogenic mutations, including 25 homozygotes and 9 compound heterozygotes. Seven patients carried one heterozygous pathogenic mutation without an identified second mutant allele (Table 1). Thus, 17.45% (41/235) of the patients had molecular defects in *GJB2*, which is similar to the ratio of typical areas in China (18.31%; χ^2^ = 0.0651, P > 0.05). Thus, *GJB2* mutant alleles accounted for 16.0% (75/470) of the total alleles in 235 NSHI patients. Of those patients, 14.45% (34/235) had confirmed molecular etiology, and 2.98% (7/235) carried only one pathogenic mutation. The most common mutant allele was c.235delC, followed by c.299_300delAT, c.512insAACG, c.139G > T and c.176del16, which accounted for 11.1% (52/470), 1.7% (8/470), 1.1% (5/470), 0.6% (3/470) and 0.6% (3/470), respectively.

**Table 1:** Genotypes of patients with mutations in the GJB2 gene

**Allele1**			**Allele2**			
**Nucleotide change**	**Consequence or amino change**	**Category**	**Nucleotide change**	**Consequence or amino change**	**Category**	**Number of patients**
c.235delC	Frameshift	Pathogenic	c.235delC	Frameshift	Pathogenic	20
c.139G > T	Glu at 47 into Stop	Pathogenic	c.139G > T	Glu at 47 into Stop	Pathogenic	1
c.176del16	Frameshift	Pathogenic	c.176del16	Frameshift	Pathogenic	1
c.299del AT	Frameshift	Pathogenic	c.299delAT	Frameshift	Pathogenic	2
c.512insAACG	Frameshift	Pathogenic	c.512insAACG	Frameshift	Pathogenic	1
c.235delC	Frameshift	Pathogenic	c.299delAT	Frameshift	Pathogenic	3
c.176del16	Frameshift	Pathogenic	c.235delC	Frameshift	Pathogenic	1
c.235delC	Frameshift	Pathogenic	c.512insAACG	Frameshift	Pathogenic	1
c.35insG	Frameshift	Pathogenic	c.512insAACG	Frameshift	Pathogenic	1
c.139G > T	Glu at 47 into Stop	Pathogenic	c.299delAT	Frameshift	Pathogenic	1
c.9G > A		Pathogenic	c.235delC	Frameshift	Pathogenic	1
c.11G > A		Pathogenic	c.235delC	Frameshift	Pathogenic	1
c.235delC	Frameshift	Pathogenic				5
c.512insAACG	Frameshift	Pathogenic				1
c.431-450del19	Novel					1

c.235delC was the hotspot in this patient cohort, which reportedly is the most prevalent mutation in East Asian and Southeast Asian populations, but is not common in European or Oceania populations [28]. Twenty patients were homozygous for the c.235delC mutation, seven were compound heterozygous with another pathogenic mutation, and five were heterozygous for the c.235delC mutation (Table 1). One novel frameshift mutation was identified, 431_450del19 (Table 1). Overall, 75 mutant alleles (including the unclassified missense variants) were identified in 41 unrelated patients. c.235delC alone accounted for 69.33% (52/75) of the total mutant alleles, compared to 71.64% (96/134) in typical areas of China [17], 69.18% (101/146) in northern China [29]. The differences were not significant among ours and other studies of the Chinese population (χ^2^ = 0.1240, χ^2^ = 0.0006, P > 0.05).

The *GJB2* mutation frequency was 16.67% (7/42) in minority patients, involving two mutations (i.e., c.235delC and c.139G > T) whose allele frequencies were 14.29% (12/84) and 2.38% (2/84), respectively. Of all the 20 homozygous c.235delC patients, three Yi, two Bai, and one Hui patient were homozygous for c.235delC. One Dai patient carried homozygous c.139G > T. The *GJB2* mutation frequency in Han Chinese patients was 17.62% (34/193), involving nine mutations: c. 235delC, c.299de1AT, c.512insAACG, c.176del16, c.35insG, c.139G > T, c.9G > A, c.11G > A, c.431_450del19. The allele frequencies were 10.36% (40/386), 2.07% (8/386), 1.30% (5/386), 0.78% (3/386), 0.26% (1/386), 0.26% (1/386), 0.26% (1/386), 0.26% (1/386) and 0.26% (1/386) in Han Chinese patients, respectively. The c.235delC allele frequency in the minority patients was not significantly different from that in the Han patients (χ^2^ = 1.0790, P > 0.05).

We analyzed the *GJB2* gene in 200 control subjects with normal hearing and found three types of deleterious mutations, c.235delC, c.299_300delAT and c.139G > T, carried by seven subjects in the heterozygous state. This suggested a *GJB2* mutation carrier rate of ~3.5% (7/200) in the general population. Meanwhile, the carrier rates of the *GJB2* mutation in Korea, Japan, Taiwan, Ashkenazi Jews and in the Midwestern United States are reported to be 2%, 2.08%, 2.55%, 4.76%, and 3.01%, respectively [5,20,21,30-32].

### mtDNA 12S rRNA

Eight and a half percent (20/235) of patients carried the *mtDNA 12S rRNA* 1555A > G mutation. Among the 20 patients, 7 had a clear history of aminoglycoside use. The frequency of 1555A > G mutation was 7.77% (15/193) in Han patients and 11.90% (5/42) in minority patients. The five minority patients consisted of two Bai, one Naxi, one Hani and one Hui. None of the 200 control subjects were found to carry the 1555A > G mutation.

### SLC26A4

Sequence analysis of the *SLC26A4* gene in these 235 patients identified nine patients with two confirmed pathogenic mutations, and five patients carried one confirmed pathogenic mutation. Six patients were compound heterozygous for one pathogenic mutation and one unclassified variant, and three patients carried one unclassified variant (Table 2). Thus, mutations in *SLC26A4* were identified in 9.97% (23/235) of patients with hearing impairment in this study.

**Table 2:** Genotypes of SLC26A4 gene-related hearing impairment in Yunnan

**Allele1**			**Allele2**				
**Nucleotide change**	**Consequence or amino change**	**Category**	**Nucleotide change**	**Consequence or amino change**	**Category**	**Number of patients**	
c.IVS7-2A > G	aberrant splicing	Pathogenic	c.IVS7-2A > G	aberrant splicing	Pathogenic	4	EVA
c.IVS7-2A > G	aberrant splicing	Pathogenic	c.IVS7-2A > G	aberrant splicing	Pathogenic	1	nl
c.IVS7-2A > G	aberrant splicing	Pathogenic	c.754 T > C	S252P	Pathogenic	1	ND
c.IVS7-2A > G	aberrant splicing	Pathogenic	c.1548insC	FX517,526X	Pathogenic	1	EVA
c.2168A > G	H723R	Pathogenic	c.1079 T > C	A360V	Pathogenic	1	EVA
c.2168A > G	H723R	Pathogenic	c.84C > A	S28R	Pathogenic	1	nl
c.IVS7-2A > G	aberrant splicing	Pathogenic	c.1975G > C	V659L	Pathogenic	1	EVA
c.IVS7-2A > G	aberrant splicing	Pathogenic	c.IVS9 + 1G > A	aberrant splicing	Pathogenic	2	EVA
c.IVS7-2A > G	aberrant splicing	Pathogenic	c.2265C > A	T755T	Silent variant	1	EVA
c.IVS7-2A > G	aberrant splicing	Pathogenic	c.IVS5 + 27 T > C	Variant in intron	Unclassified variant	1	EVA
c.1548insC	FX517,526X	Pathogenic	c.IVS11 + 47 T > C	Variant in intron	Unclassified variant	1	EVA
c.2168A > G	H723R	Pathogenic				1	EVA
c.IVS7-2A > G	aberrant splicing	Pathogenic				3	nl
c.IVS7-2A > G	aberrant splicing	Pathogenic				1	EVA
c.intron6-75_ 76 insTGTG	aberrant splicing	Unclassified variant				1	nl
c.IVS7 + 35A > G	Variant in intron	Unclassified variant				1	nl
c.IVS7 + 40G > A	Variant in intron	Unclassified variant				1	nl
WT			WT			2	EVA

*nl*, normal; *EVA*, enlarged vestibular aqueduct; *IVS7*, intravening sequence7(intron 7); *IVS9*, intravening sequence9(intron 9); *IVS5*, intravening sequence5(intron 5); *IVS11*, intravening sequence11(intron 11); *WT*, wildtype.

A total of seven different pathogenic mutations (IVS7-2A > G, c.2168A > G, c.84C > A, c.1975G > C, c.754 T > C, c.1548insG, IVS9 + 1G > A) and six unclassified variants (intro6-75_76insTGTG, IVS11 + 47 T > C, c.2265C > A, IVS5 + 27 T > C, IVS7 + 35A > G, IVS7 + 40G > A) were found (Table 2). The c.1548insG mutation resulted in a premature stop codon and a truncated protein with only 526 amino acid residues. For those mutations, IVS7-2A > G, c.2168A > G, c.84C > A, c.1975G > C, c.754 T > C and IVS9 + 1G > A, were previously reported in patients with hearing loss [33-38]. For other variants, IVS11 + 47 T > C, c.2265C > A, IVS5 + 27 T > C, IVS7 + 35A > G, IVS7 + 40G > A and intro6-75-76insTGTG, the NNSPLICE software, available at http://www.fruitfly.org/seq_tools/splice.html was used for analysis. The results indicated that those variants did not predict gain or loss of a splice site with this variant, and those variants were considered benign. Thus, *SLC26A4* mutant alleles accounted for 82.61% (38/46) of the total alleles in 235 NSHI patients. For those patients, 43.48% (10/23) had a confirmed molecular etiology, and 43.48% (10/23) carried only one pathogenic mutation.

IVS7-2A > G was the hotspot mutation region in this patient cohort, for which five patients were homozygous, six were compound heterozygous, and four were heterozygous (Table 2). One of the four patients with the heterozygous IVS7-2A > G mutation was also homozygous for the *GJB2* c.235delC mutation. Thus, this patient may be only an IVS7-2A > G carrier, with defects in *GJB2* being the main cause of hearing loss. The IVS7-2A > G mutation accounted for 64.63% (53/82, counting only the definite pathogenic and most likely pathogenic variants) of all *SLC26A4* mutant alleles in this population.

The *SCL26A4* mutation frequency was 9.52% (4/42) in minority patients, involving six mutations: IVS7-2A > G, c.intron6-75_76insTGTG, c.2168A > G, c.84A > G, c.IVS11 + 47 T > C and c.1548insG. One Hui carried IVS7-2A > G/IVS7-2A > G, one Yi carried IVS7-2A > G/1548insC, one Bai carried IVS7-2A > G/IVS5 + 27 T > C and one Zhuan carried IVS11 + 47 T > C/1548insC. The allele frequencies were 2.38% (2/84), 1.19% (1/84), 1.19% (1/84), 1.19% (1/84), 1.19% (1/84) and 1.19% (1/84) for IVS7-2A > G, c.intron6-75_76insTGTG, c.2168A > G, c.84A > G, IVS11 + 47 T > C and c.1548insG, respectively. For the Han Chinese population, *SLC26A4* mutation frequency was 9.84% (19/193), involving 11 mutations: IVS7-2A > G, c.2168A > G, c.1079 T > C, c.1975G > C, c.754 T > C, IVS9 + 1G > A, IVS11 + 47 T > C, c.2265C > A, IVS5 + 27 T > C, IVS7 + 35A > G and IVS7 + 40G > A. The allele frequencies were 6.64% (19/286) for IVS7-2A > G, 1.05% (3/286) for c.2168A > G, 0.70% (2/286) for IVS11 + 47 T > C and 0.35% (1/286) for each else.

### Temporal bone CT scan

A total of 100 cases, comprised of 16 cases with *SLC26A4* mutations (12 Han Chinese patients and four minority patients) and 84 control cases without any *SLC26A4* mutations or variants (37 minority cases and 47 Han Chinese patients), were examined by temporal bone CT scan. Bilateral EVA was detected in 16 patients (14 patients with *SLC26A4* mutation, 2 without *SLC26A4* mutation) (Table 2). Four patients carried a homozygous IVS7-2A > G mutation, five carried a compound heterozygous IVS7-2A > G/1548insC, 2168A > G/84C > A, IVS7-2A > G/IVS9 + 1G > A and IVS7-2A > G/1975G > C, and five carried only one pathogenic mutation(IVS7-2A > G, 1548insC and 2168A > G); another two were from the minority control patients without any *SLC26A4* mutations or variants. Temporal CT scan results were normal in the remaining patients (Table 2).

Regarding race, six minority patients (one Hui, one Bai, one Zhuang, and three Yi) presented with EVA. Four (4/42, 9.52%) of six patients carried a *SLC26A4* mutation, whose genotypes were: IVS7-2A > G/IVS7-2A > G, IVS7-2A > G/IVS5 + 27 T > C, IVS11 + 47 T > C/1548insC, 2168A > G/84C > A. The EVA ratio in the Chinese deaf population was at least 11%, and that in patients of Han ethnicity reached at least 13%.

## Discussion

Many hearing loss-related gene mutations have been described to date, most of which are isolated mutations in some populations. In this study, mutation analysis was performed in 235 patients with moderate to profound sensorineural hearing loss. A total of 35.74% deaf patients showed evidence of genetic involvement based on either genetic screening or family history, and 17.45%, 9.79%, and 8.51% of the patients were determined to have inherited hearing impairment caused by *GJB2*, *SLC26A4*, and *mtDNA 1555A > G* mutations. *GJB2* mutation was detected in 16.67% (7/42) of minority patients and 17.62% (34/193) of Han Chinese patients. *SLC26A4* mutation was found in 9.52% (4/42) of minority patients and 9.84% (19/193) of Han Chinese patients. The frequencies of *mtDNA 12S rRNA* mutation in minority and Han Chinese patients were 11.90% (5/42) and 7.77% (15/193). There was no significant difference in deafness associated gene mutational spectrum or frequency between the Yunnan minority patients and the Han patients (Table 3). These results will facilitate effective risk assessment and genetic counseling for hearing loss patients and their families in Yunnan.

**Table 3:** Prevalence of GJB2, SLC26A4 and mtDNA 1555A > G mutation in the Asian

**Different areas**	**Patients’ number**	**Prequency of pathologic variants (%)**			**References**
		**GJB2**	**SLC26A4**	**mtDNA 1555A > G**	
Typical areas of China	284	18.31%	13.73%	1.76%	Yuan YY et al. [17]
		(52/284)	(39/284)	(5/284)	
Yunnan	42	16.67%	9.52%	11.90%	This study
(minority)		(7/42)	(4/42)	(5/42)	
Yunnan	193	17.62%	9.84%	7.77%	This study
(Han Chinese)		(34/193)	(19/193)	(15/193)	
Tibet	114			1.75%	Yuan YY et al. [40]
(Tibetan)				(2/114)	
Xinjiang	199	9.05%	2.01%	1.51%	Chen Y et al. [41]
(Uyghur)		(18/199)	(4/199)	(3/199)	
Korea	1256	1.91%			Kim SY et al. [42]
		(24/1256)			
Japan	1343	14.2%			Tsukada K et al. [43]
		(1911/1343)			

### GJB2

Sequencing of the *GJB2* gene coding region revealed that 14.45% (34/235) of the patients carried two pathogenic *GJB2* mutations and 2.98% (7/235) carried only one mutant allele. There was no significant difference between our results and the report of Dai (χ^2^ = 0.0285, χ^2^ = 3.6975, P > 0.05) of 2,063 unrelated NSHI students from 23 regions of China (14.9% had two pathogenic *GJB2* mutations and 6.1% carried only one mutant allele) [39].

The 235delC mutation was markedly associated with the risk of NSHL in East Asian and Southeast Asian populations, but not significantly in European or Oceanian populations [28]. In our study, the most common mutation was c.235delC, followed by c.299delAT. The common Caucasian mutation c.35delG was not found. Two mutations, c.235delC and c.299delAT, accounted for 80.00% (60/75) of the *GJB2* mutations in our patients, 97% in a Taiwanese population, and 91% in another Chinese population.

The 235delC allele frequency in the Yunnan minority patients (14.29%) was not significantly different from that in the Han patients (10.36%, χ^2^ = 1.0790, P > 0.05), but was higher than that in Tibetan (1.28%,χ^2^ = 78.2067, P < 0.05) [40], Uyghur (3.52%, χ^2^ = 227.6810, P < 0.05) [41], Korean (0.44%, χ^2^ = 116.7899, P < 0.05) [42], and Japanese patients (5.29%, χ^2^ = 458.1409, P < 0.05) [43].

c.512insAACG and c.9G > A were first reported in China by Dai. c.512insAACG is a pathogenic alteration with an allele frequency of 0.58% (12/2,063) in the Chinese population. In our study, three patients carried c.512insAACG (one homozygote, two compound heterozygotes, and one heterozygote). c.11G > A accounted for 0.43% (1/235) in Yunnan, 10.6% of Taiwanese *GJB2*[7,31]. c.9G > A, which was a nonsense mutation, carried by one patient 0.43% (1/235) in Yunnan.

In addition to these mutations, we found a novel frameshift mutation, c.431_450del 19, in a heterozygous patient. This mutation was not detected in the control group. The mutation resulted in a premature stop codon. It is highly likely to be pathogenic. Regretfully, we failed to take the history and blood sample from the family members of the patient, so it is not clear whether c.431_450del 19 represents an autosomal dominant mutation or is an autosomal recessive with an unidentified second mutant allele in either the same gene or in a different gene. To clarify the exact roles of the mutation, careful follow-up and functional studies are needed.

### SLC26A4

Mutations in *SLC26A4*, which encodes Pendrin, are a common cause of deafness, and responsible for both syndromic and nonsyndromic hearing loss. The *SLC26A4* mutation spectrum varies widely among ethnic groups. In our study population, mutations in *SLC26A4* were identified in 9.79% (23/235) of individuals, which was lower than that in typical areas of China (18.66%, χ^2^ = 8.1030, P < 0.05), but higher than that in Uyghur patients (2.01%, χ^2^ = 11.1711, P < 0.05) [41]. In addition, prevalent mutations in different ethnic groups are very different. The *SLC26A4* mutational spectrum in Yunnan is similar to that reported in the overall Chinese population, with IVS7-2A > G being the hotspot mutation. This mutation spectrum was different from that in Japanese, Korean, northern European and Danish populations. In Japan, H723R is the most prevalent mutation. In Korea, IVS7-2A > G and H723R are the two most prevalent mutations [44,45]. There seems to be a shift in mutation from IVS7-2A > G to H723R from China to Japan, with Korea in the middle. This observation suggests that IVS7-2A > G and H723R mutations may be ancient mutations in China and Japan, respectively. The unique rare mutations evolved more recently. A recent study of 100 unrelated patients with EVA in European Caucasians by Albert et al. revealed a diverse mutation spectrum without prevalent mutations; only 40 patients carried *SLC26A4* mutations [46]. It is not clear why the mutations in *SLC26A4* account for a much lower percentage of patients with EVA in Caucasian patients. Presumably, other genetic factors and environmental factors are involved in the pathogenesis of EVA in Caucasians. A recent study of 109 unrelated probands with EVA in a Danish population revealed that the most frequent mutation was 1246A > C. This implies that Danish and Chinese populations are of different ancestry [47].

In our study population, the frequency of SCL26A4 mutation was 9.52% in minority patients, which was not different from that of Han patients (9.84%; χ^2^ = 0.0498, P > 0.05). The allele frequencies of hotspot mutation, IVS7-2A > G, were 2.38% and 6.64% for minority and Han populations, respectively. The difference may due to the relatively small sample size.

It is interesting to note that eight patients with inner ear malformations carried one pathogenic mutation, and two patients with inner ear malformations carried no pathogenic mutation in *SLC26A4*. If *SLC26A4* defects are the cause of the phenotype of inner ear malformation, pathogenic mutations may be located in introns or promoter regions that were not sequenced. Unknown genes or even environmental factors may contribute to the malformation.

### mtDNA 12S rRNA

*MtDNA 12S rRNA* A1555G mutation was detected in nearly 8.51% (20/235) of our nonsyndromic hearing impaired population. The mutation in minority patients was not different from that of Han patients. However, the mutation rate in Yunnan deaf population was much higher than that in typical areas of China (1.76%) according to Yuan et al. This mutation is found in approximately 3% of Japanese, 0.5–2.4% of Caucasian living in Europe or America and 5.3% of Indonesian sensorineural deafness patients [48]. But close to the Guo’ research in northern China (8.56% 44/514) [29]. This is probably due to two reasons. First, Yunnan is in the southwest of China, where the overall quality of medical care is lower than the average in China; aminoglycoside antibiotic abuse exists. Second, there may be a founder effect. Northern Chinese are derived largely from the Mongolian lineage, which is quite different from the ancestor(s) of Caucasians in European countries.

Our results showed no significant difference in common deafness genes’ mutational frequencies or spectrum between the minority patients and the Han patients from Yunnan Province (χ^2^ = 0.3189, P > 0.05). However, the number of individuals included from the non-Han population may have been too small to draw any conclusions. On the basis of our findings, we recommend that elective genetic testing be performed in newborns, pregnant females, or patients requiring the administration of aminoglycoside antibiotics to prevent hearing loss in Yunnan province.

## Conclusion

A total of 35.74% deaf patients showed evidence of genetic involvement, based on either genetic screening or family history, and 17.45%, 9.79%, and 8.51% of these patients were determined to have inherited hearing impairment caused by *GJB2*, *SLC26A4*, and mtDNA 1555A > G mutations. Our study showed that there was no significant difference between Yunnan minority patients and the Han patients in the deafness-associated gene mutation spectrum. Our study provides much-needed information regarding deafness molecular epidemiology of the Yunnan province, providing a rationale for deafness gene diagnosis in Yunnan province.

## Abbreviations

CT: Computed tomography; NSHL: Nonsyndromic hearing loss; EVAs: Enlarged vestibular aqueducts; mtDNA: Mitochondrial DNA; PCR: Polymerase chain reaction; DHPLC: Denaturing high-pressure liquid chromatography.

## Competing interests

The authors declare that they have no competing interests.

## Authors’ contributions

FX, YY, and XD carried out the molecular genetic studies and participated in sequence alignment. FX and YY drafted the manuscript. XG and GW carried out temporal CT scan and thyroid hormone assays. JZ, FY and MH participated in sequence alignment and performed the statistical analyses. JL and DH participated in the design of the study. PD and YY conceived the study, participated in its design and coordination, and helped draft the manuscript. All authors have read and approved the final manuscript.
